# Exosomal CNP and CNP-Related microRNAs: An Open Window into Brugada Syndrome?

**DOI:** 10.3390/biomedicines14051094

**Published:** 2026-05-12

**Authors:** Manuela Cabiati, Federico Vozzi, Elisa Persiani, Marcello Piacenti, Andrea Rossi, Agnese Sgalippa, Antonella Cecchettini, Gianluca Solarino, Giulio Zucchelli, Lorenzo Mazzocchetti, Pasquale Notarstefano, Letizia Guiducci, Maria Aurora Morales, Silvia Del Ry

**Affiliations:** 1 Laboratory of Biochemistry and Molecular Biology, Institute of Clinical Physiology, National Research Council (CNR), 56100 Pisa, Italy; manuela.cabiati@cnr.it (M.C.); federico.vozzi@cnr.it (F.V.); elisa.persiani@cnr.it (E.P.); agnese.sgalippa@santannapisa.it (A.S.); antonella.cecchettini@unipi.it (A.C.); letizia.guiducci@cnr.it (L.G.); auroramorales@icloud.com (M.A.M.); 2 Fondazione Toscana Gabriele Monasterio, 56100 Pisa, Italy; marcello.piacenti@ftgm.it (M.P.); andrea.rossi@ftgm.it (A.R.); 3 Health Science Interdisciplinary Center, Sant’Anna School of Advanced Studies, 56100 Pisa, Italy; 4 Department of Clinical and Experimental Medicine, University of Pisa, 56100 Pisa, Italy; 5 Cardiology Department, Versilia Hospital, 55041 Lido di Camaiore, Italy; gianluca.solarino@uslnordovest.toscana.it; 6 Second Division of Cardiology, Azienda Ospedaliero Universitaria Pisana, 56100 Pisa, Italy; zucchelli76@gmail.com (G.Z.); info@aritmologomazzocchetti.it (L.M.); 7 Cardiovascular and Neurological Department, San Donato Hospital, 52100 Arezzo, Italy; pasqualenotarstefano@gmail.com

**Keywords:** exosomal microRNAs, C-type natriuretic peptide, mRNA, Brugada Syndrome, Droplet Digital™ PCR

## Abstract

**Background:** Brugada Syndrome (BrS) is a cardiac arrhythmia associated with an increased risk of ventricular arrhythmias and sudden cardiac arrest. Although the arrhythmic substrate is traditionally localized to the ventricles, atrial fibrillation (AF) is frequently observed, suggesting a shared molecular substrate between atrial and ventricular arrhythmias. C-type natriuretic peptide (CNP) and related microRNAs (miRNAs) modulate atrial and ventricular physiology, but their roles in exosomes in BrS have not been investigated. **Objectives:** To investigate alterations in *CNP* mRNA expression and changes in the expression of selected *CNP*-associated miRNAs implicated in AF, both analyzed in exosomes isolated from individuals with BrS and from healthy controls. **Methods:** Exosomes were isolated from the plasma of BrS patients without a history of overt AF and from healthy controls. In silico analyses identified *CNP*-targeting miRNAs implicated in AF. Exosomal *CNP* and *CNP*-related miRNAs were analyzed using Droplet Digital PCR. **Results:** BrS patients exhibited a significant increase in exosomal *CNP* mRNA expression levels compared with controls. MiR-138-5p was selectively downregulated, whereas other AF-related *CNP*-targeting miRNAs (miR-4443, miR-206, miR-142-5p, miR-223-5p) showed comparable levels between groups. A positive correlation between exosomal *CNP* and miR-223-5p and miR-4443 suggests shared regulatory pathways. **Conclusions:** these findings indicate that exosomal profiling may provide a more sensitive approach than conventional circulating measurements to detect molecular remodeling in BrS. The observed alterations highlight a potential shared molecular substrate between atrial and ventricular arrhythmias and may inform future studies aimed at refining diagnostics and developing targeted therapeutic strategies.

## 1. Introduction

### 1.1. The Connection Between Brugada Syndrome and Atrial Fibrillation

**Figure 1:**
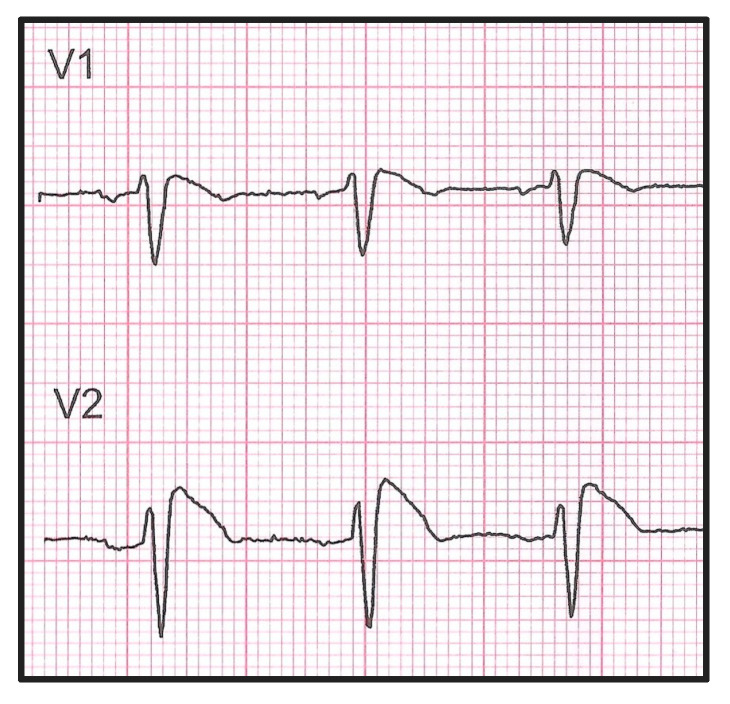
Representative electrocardiogram of a patient with Brugada Syndrome (BrS) showing the typical type 1 ECG pattern. V1 and V2 are the precordial (chest) leads in 12-lead ECG.

Brugada Syndrome (BrS) is a type of cardiac arrhythmia associated with an elevated risk of dangerous ventricular arrhythmias and unexpected cardiac arrest [1,2]. Although the arrhythmic substrate in BrS has traditionally been localized to the ventricles, emerging data indicate that atrial fibrillation (AF) may also be a clinical manifestation of the condition, with prevalence rates reported between 6% and 38% [3,4]. A meta-analysis by Kewcharoen et al. found that the presence of AF significantly increases the likelihood of major arrhythmic events in individuals with BrS [5]. It is well known that BrS is primarily caused by mutations in the SCN5A gene [6], a member of the voltage-gated sodium channel gene family that plays an essential role in regulating the heart’s electrical activity. Alterations in SCN5A are not exclusive to BrS; they have also been associated with a range of other cardiac rhythm disorders, including atrial arrhythmias, ventricular arrhythmias, long QT syndrome type 3, and progressive conduction system disease [1,2] (Figure 1).

Among the atrial arrhythmias connected to SCN5A mutations are atrial fibrillation and atrial standstill. These genetic abnormalities can promote ectopic electrical activity, prolong atrial action potential duration, and heighten atrial excitability, thereby increasing susceptibility to atrial fibrillation [7,8].

### 1.2. Signaling Patterns of C-Type Natriuretic Peptide Regulation

Atrial fibrillation can be triggered by dysregulation of atrial electrophysiology and calcium handling, processes that are influenced by β-adrenergic receptor (β-AR) activation via cAMP-mediated pathways, especially when β-AR signaling is abnormally increased [9]. In a recent study of ours [10], we observed, for the first time, the expression of C-type natriuretic peptide (*CNP*; encoded by the *NPPC* gene) mRNA in BrS using Droplet Digital™ PCR (ddPCR) technology, demonstrating its presence and activation in individuals with this condition. The natriuretic peptides, a group of hormones to which CNP belongs, are known for their cardioprotective effects and key role in maintaining cardiovascular balance; they are produced in the atrial myocardium, where they exert localized actions within the heart [11,12]. CNP circulates at low concentrations, indicating its primary function is likely through local paracrine signaling. It uniquely activates the natriuretic peptide receptor B (NPR-B), a guanylyl cyclase (GC) receptor that, upon activation, elevates intracellular cGMP levels [13]. Atrial fibrosis is a well-recognized clinical feature of atrial fibrillation and an essential process in the development of arrhythmia, where microRNAs (miRNAs) are involved in both physiological and pathophysiological regulation [14,15]. They are short, non-coding RNAs that primarily bind to the 3′ untranslated region (3′UTR) of target mRNAs, leading to their degradation or translational repression [16,17]. Consequently, they offer valuable complementary insights into the regulation of AF mRNA signature and have emerged as promising therapeutic targets, with synthetic miRNA mimics and inhibitors being explored as potential treatment strategies. Moreover, with rapid advances in molecular biology and biotechnology, increasing evidence highlights the critical role of exosomes, particularly exosomal miRNAs, in the pathophysiology of cardiovascular diseases. Several cardiovascular-relevant cell types, including mast cells, endothelial cells, macrophages, and mesenchymal stem cells, are known to actively produce and release exosomes [18,19,20,21]. Both exosomes and miRNAs are abundantly distributed across various tissues and biological fluids, with circulating miRNAs primarily transported in exosomal form [22,23,24]. Exosomal miRNAs participate in the regulation of cardiovascular diseases through multiple interconnected molecular pathways, including apoptosis, inflammation, autophagy, fibrosis, and vascular regeneration, highlighting their relevance as potential therapeutic targets [25,26,27,28,29].

To better elucidate the potential involvement of CNP in BrS, this study aims to investigate alterations in the expression of *CNP* as well as changes in the expression of selected *CNP*-associated miRNAs implicated in AF, both analyzed in exosomes isolated from individuals with BrS and healthy controls.

In brief, the specific objectives of this study are to:

(a) Analyze *CNP* system expression variation in exosomes obtained from BrS patients and healthy controls using ddPCR;
(b) Perform a comprehensive in silico analysis to identify miRNAs targeting the *CNP* gene using bioinformatics algorithms;
(c) Identify *CNP*-related miRNAs previously associated with atrial fibrillation;
(d) Characterize and compare the exosomal profiles of these *CNP*-associated miRNAs in BrS patients and healthy controls by ddPCR.

## 2. Materials and Methods

### 2.1. Subjects Enrollment and Plasma Collection

As previously described in our earlier work [10], this study is a multicenter, retrospective, non-randomized, and non-profit investigation analysis including BrS patients and controls. It was conducted in compliance with the Declaration of Helsinki and was approved by the local ethics committee, “Comitato Etico Regionale per la Sperimentazione Clinica della Regione Toscana Sezione: AREA VASTA NORD OVEST” (Fondazione Toscana CNR/Regione Toscana per la Ricerca Medica e di Sanità Pubblica, Mod C1 Vers 2016011, consent approval date: 5 November 2020, nr. 18542) [30]. The diagnosis of BrS was based on the 2015 European Society of Cardiology guidelines for the management of ventricular arrhythmias and the prevention of sudden cardiac death [31]. Eligibility criteria included individuals aged 14 to 65 years presenting with a spontaneous type 1 BrS ECG [30,31,32,33]. The control group consisted of age-matched individuals undergoing routine outpatient cardiology evaluations, all of whom exhibited normal resting ECGs. Subjects were excluded if they had structural heart disease, were pregnant, had coronary artery disease, severe renal or hepatic dysfunction, other medical conditions that might compromise protocol adherence, or if informed consent was not obtained. Structural heart disease was excluded in all participants prior to enrolment using non-invasive cardiac imaging modalities, such as echocardiography and/or cardiac magnetic resonance imaging (MRI). We studied 32 subjects, including 14 controls (C) and 18 subjects with spontaneous type 1 BrS without a history of overt AF. Both BrS patients and controls underwent baseline ECGs using standard recording parameters (paper speed 25 mm/s, amplification 10 mm/mV, and a sampling rate of 10 s at 500 Hz). For molecular biology studies, exosomes were obtained from blood samples of BrS and control subjects.

### 2.2. In Silico Analysis

As also reported in a previous study of ours [34], an in silico analysis requires the use of bioinformatics algorithms such as TargetScan (http://www.targetscan.org/vert_72/) URL (accessed on 26 March 2026) and PicTar (https://pictar.mdc-berlin.de/) URL (accessed on 26 March 2026) to predict the biological targets of microRNAs. They are based on alignment programs that identify preserved sequences of 7–8 nucleotides with high complementarity of the miRNA “seed sequences” and high thermodynamic stability, with scores ranging from 0 to 100, where 0 represents no complementarity, and 100 represents perfect complementarity. miRNA-targeted genes are networked using DIANA tools (http://diana.imis.athena-innovation.gr/DianaTools/index.php?r=microT_CDS/index) URL (accessed on 26 March 2026), a software based on an automatically selected weighting method to recognize a number of co-expressed genes relevant to target genes, making the regulatory networks more complete. They are validated with DIANA MicroT (DIANA-microT-ANN). In the present study, the prediction of miRNA/mRNA interactions, to obtain accurate results decreasing the false-positive rates, was challenging due to the relatively limited knowledge of the *CNP* gene structure; our analysis weighted the biological aspects used by TargetScan 7.2 and DIANA microT 2023 prediction tools, taking advantage of their algorithm.

### 2.3. Exosome Isolation and Characterization, Vesicular RNA Extraction and Reverse Transcription

As reported in our previous study [35], exosomes were isolated from 600 μL of plasma using a dedicated and innovative assay (exoRNeasy midi kit, QIAGEN GmbH, Hilden, Germany) and the reliability of the isolation method was validated through transmission electron microscopy (TEM), used to assess exosome morphology, and by Western blotting analysis, performed to confirm the presence of specific exosomal proteins, before RNA extraction. In addition, quantitative and size distribution characterization of isolated vesicles was performed using nanoparticle tracking analysis (NTA, NanoSight Ltd., Amesbury, UK), equipped with a405nmlaser and NTA2.3 analytic software, confirming both vesicle concentration (1.72 × 10^10^ exosomes/mL) and size profile.

Plasma vesicles were lysed using a phenol/guanidine-based reagent (QIAzol, QIAGEN, Hilden, Germany), and the lysate was collected by centrifugation. After chloroform addition and phase separation, the aqueous phase was recovered, mixed with ethanol, and applied to a RNeasy MinElute spin column to allow RNA binding. Following washes with dedicated buffers to remove contaminants, exosomal RNA was eluted in 16 μL of RNase-free water and stored at −80 °C. RNA integrity, purity, and concentration were assessed by measuring absorbance at 260 and 280 nm (NanoDrop Thermofisher, Waltham, MA, USA) and applying the Beer–Lambert law with expected values between 1.8 and 2.1, for protein contamination. Exosomal miRNA reverse transcription was performed using an all-in-one cDNA Synthesis Kit (Takara Bio Inc., Kusatsu, Shiga, Japan) that provides a comprehensive solution for the efficient synthesis of first-strand miRNA cDNA from RNA samples (Mir-X miRNA First-Strand Synthesis Kit, Takara, Takara Bio Inc., Kusatsu, Shiga, Japan). The reverse transcription was performed in a thermocycler (My Cycler, (Bio-Rad Laboratories, Hercules, CA, USA) with an incubation step for 1 h at 37 °C; then the reaction was stopped by heating the mixture for 5 min at 85 °C, to inactivate the enzymes, prior to a final hold at 4 °C.

### 2.4. Droplet Digital™ PCR Quantification

As also reported in a previous study of ours [10] for *CNP* mRNA expression, the cDNA diluted 1:2 (12.5 ng/μL) was added to ddPCR Eva Green Supermix (Bio-Rad Laboratories, Hercules, CA, USA) along with *CNP* pre-cust primers (Hs_*NPPC_*2_SGQuantitect Primer Assay, QIAGEN, Milan, Italy). For miRNA expression, the mature miRNA sequences used as forward primers were downloaded from the miRBase database (www.mirbase.org, access date 7 October 2025) (Table 1) and synthesized by Sigma Aldrich (Milan, Italy).

EvaGreen-based droplet digital PCR was performed for miRNAs using a cDNA pool from human exosomal samples, and the assays were optimized accordingly. The experiment was performed at two different temperatures (Ta = 55 °C and 58 °C) and at three different cDNA dilutions (1:20, 1:100, and 1:200) and miRNA primer concentrations (100, 150, 200, and 300 nM). Briefly, 22 µL of PCR reaction, containing 100–300 nM of primers, 5 µL of diluted cDNA, 11 µL of 2× EvaGreen Supermix (Bio-Rad, Laboratories, CA, USA), and nuclease-free water up to the final volume, were loaded into the droplet generator cartridge (Bio-Rad Laboratories, Hercules, CA, USA). Then, 70 µL of droplet generation oil for EvaGreen (Bio-Rad Laboratories, Hercules, CA, USA) was added to specific wells in the cartridge. The cartridge was transferred to the QX200 droplet generator (Bio-Rad Laboratories, Hercules, CA, USA). After droplet generation, the droplets (≈40 µL) were loaded into a 96-well PCR plate (Bio-Rad Laboratories, Hercules, CA, USA), and the plate was heat-sealed with specific aluminum foil (Bio-Rad Laboratories, Hercules, CA, USA). Thermal cycling conditions were as follows: 95 °C for 10 min, then 42 cycles of 94 °C for 30 s and 55/57 °C for 1 min (ramping rate reduced to 1.6 °C/s), and three final steps at 4 °C for 15 min, 98 °C for 10 min, and a 4 °C indefinite hold to enhance dye stabilization. The measurement of positive and negative droplets was carried out using the QX200™ Droplet Reader (Bio-Rad Laboratories, Hercules, CA, USA). The absolute copy count was calculated based on the droplet count using the Poisson distribution and the Quanta-Soft software (Bio-Rad Laboratories, Hercules, CA, USA; version 1.7.4.0917). The dMIQE guidelines, which summarize the essential information for reporting ddPCR experiments, were followed [36].

### 2.5. Statistics

In the ddPCR method, expression values were obtained as copies/µL for each sample using QXManager software version 2.1 (Bio-Rad, Hercules, CA, USA) for absolute quantification of target genes and subsequently reported as copies per 600 µL of total exosome-derived plasma. Statistical analyses were performed using Statview 5.0.1 (Windows Statistical, SAS Institute, Inc., Cary, NC, USA). Skewed variables were log-transformed before analysis. A Student’s unpaired *t*-test was used to obtain results, expressed as mean ± SEM; *p* < 0.05 was considered significant, and relationships between variables were evaluated using linear or multivariate logistic regression.

## 3. Results

### 3.1. BrS Patient’s Characterization

Among the subjects actually included in the final analysis, the age range was 24–68 years in the BrS group and 21–70 years in the control group. Table 2 reports the clinical and anthropometric characteristics of both BrS and C.

### 3.2. In Silico Analysis of CNP-Related miRNAs

As highlighted in a previous study of ours [34], in silico analysis identified 78 miRNAs specifically targeting the human *NPPC* gene (Table 3), using a threshold of 0.7, corresponding to high sensitivity in Homo sapiens. Among the 78 predicted miRNAs targeting *NPPC*, a further prioritization step was applied based on literature evidence. MiRNAs with previous involvement in atrial fibrillation-related pathways, including atrial remodeling, fibrosis, inflammation, and cardiac electrophysiology, were selected for downstream experimental validation. We analyzed miR-4443 [context++ score (percentile) −0.29 (94); context++ score miTG score 0.77], miR-138-5p [context++ score (percentile) −0.46 (97); miTG score 0.97], and miR-206 [context++ score (percentile) −0.25 (93); miTG score 0.78], as well as miR-142-5p miR-142-5p (miTG score 0.78), and miR-223-5p, [context++ score (percentile) −0.38 (97); miTG score 0.72], which are also known to be associated with atrial fibrillation [37,38,39,40].

### 3.3. Biomolecular Analysis: Droplet Digital PCR Results

**Figure 2:**
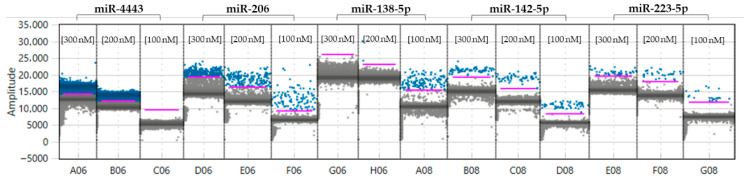
miR-4433, miR-206, miR-138-5p, miR-142-5p, miR-223-5p expression at different primer concentrations 100-200-300 nM at Ta = 55 °C using a representative healthy control sample as an example.

Based on preliminary optimization experiments for ddPCR, an annealing temperature of 55 °C with primer concentrations ranging between 100 and 200 nM, which provided the best balance between signal quality and assay performance, were selected. In particular, the final concentrations used were 100 nM for miR-138-5p; 150 nM for miR-206, miR-142-5p and miR-223-5p; and 200 nM for miR-4443 (Figure 2).

**Figure 3:**
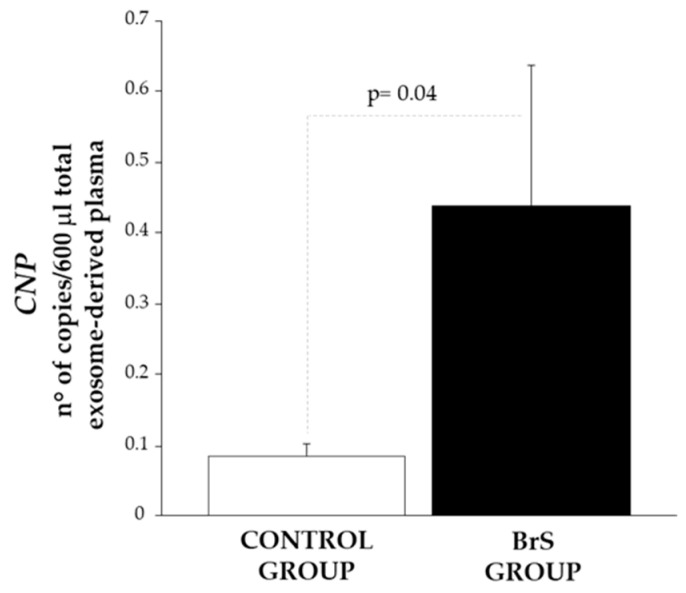
Expression level of exosomal *CNP*, in the control group (white bar) and the BrS group (black bar) (C, n = 14; BrS, n = 18; *p* = 0.05).

We then investigated whether *CNP* mRNA transcripts and a subset of *CNP*-specific miRNAs associated with atrial fibrillation (miR-4443, miR-138-5p, miR-206, miR-142-5p, miR-223-5p) were transported by circulating exosomes, and whether their expression profiles differed between patients with Brugada syndrome and healthy controls. Using ddPCR, *CNP* mRNA and all analyzed miRNAs were detectable in circulating exosomes from both BrS patients and healthy individuals. Exosomal *CNP* mRNA expression levels were significantly higher in BrS patients compared to controls (Figure 3).

**Figure 4:**
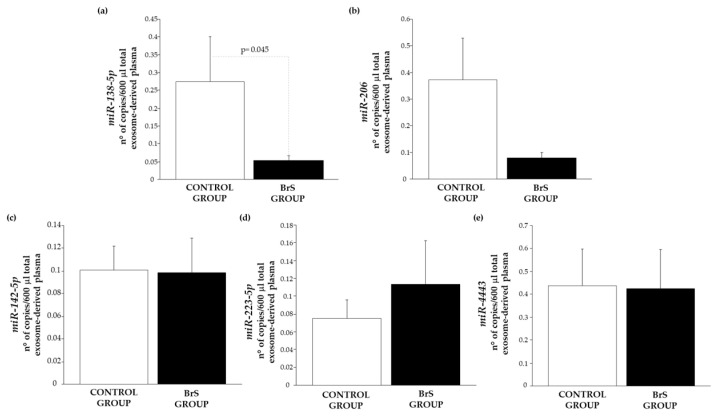
Exosomal (**a**) miR-138-5p, (**b**) miR-206 expression level in the control group (white bar) and the BrS group (black bar), (**c**) miR-142-5p, (**d**) miR-223-5p, (**e**) miR-4443 expression level in the control group (white bar) and the BrS group (black bar). (C, n = 14; BrS, n = 18).

Among the miRNAs, only miR-138-5p was significantly reduced in BrS patients (Figure 4a), while miR-206 showed a non-significant trend toward lower levels (Figure 4b). Levels of miR-142-5p, miR-223-5p, and miR-4443 were similar between groups (Figure 4c–e).

**Figure 5:**
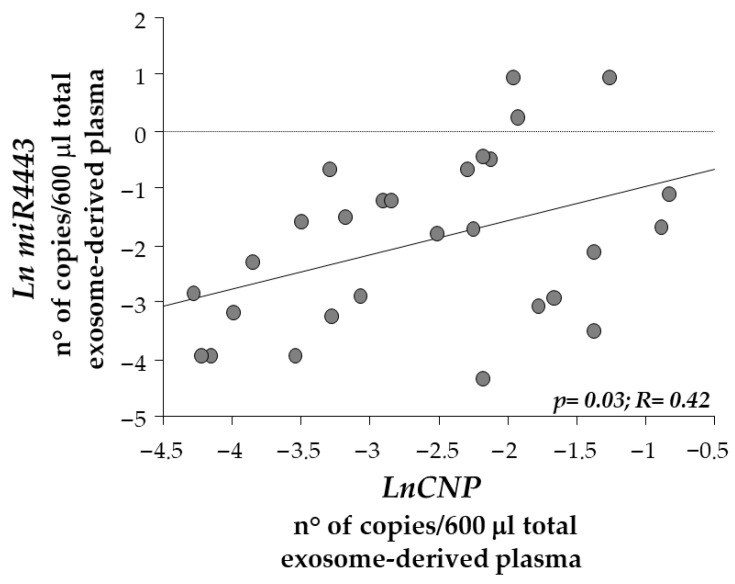
Correlation between exosomal CNP and exosomal miR-4443 (C, n = 14; BrS, n = 18; *p* = 0.05).

A positive correlation was also observed between *CNP* mRNA expression levels and miR-223-5p and miR-4443 (Figure 5). Significant correlations were observed between miR-138-5p and miR-206 (*p* < 0.0001, R = 0.89), miR-223-5p and miR-206 (*p* = 0.002, R = 0.61), miR-4443 and miR-206 (*p* = 0.0004, R = 0.62), miR-223-5p and miR-138-5p (*p* = 0.05, R = 0.48), miR-4443 and miR-138-5p (*p* = 0.005, R = 0.57), miR-223-5p and miR-142-5p (*p* = 0.006, R = 0.54), miR-4443 and miR-142-5p (*p* = 0.03, R = 0.38), miR-4443 and miR-223-5p (*p* < 0.0001, R = 0.77).

## 4. Discussion

In this study, we provide the first evidence that *CNP* mRNA transcripts and selected *CNP*-related miRNAs associated with atrial fibrillation are transported by circulating exosomes in patients with Brugada Syndrome. Importantly, our findings reveal a significant increase in exosomal *CNP* mRNA expression in BrS patients compared with healthy controls. This result diverges from our previous observations in the whole blood, where *CNP* mRNA expression levels were slightly but not significantly lower in BrS individuals [10]. This discrepancy may reflect differential compartmentalization of *CNP* in BrS, with a preferential shift toward exosomal packaging rather than remaining distributed throughout the whole blood. Alternatively, the higher detectability of *CNP* within exosomes may indicate that vesicular analysis represents a more sensitive approach for capturing disease-related molecular signals. Such a mechanism may represent an active cellular response aimed at modulating local or systemic signaling pathways, potentially reflecting early molecular remodeling in BrS even in the absence of overt structural heart disease.

These findings should be interpreted in the context of the recognized clinical association between Brugada syndrome and atrial fibrillation. AF has been reported in up to 38% of BrS patients and has been associated with a higher incidence of major arrhythmic events [3]. This supports the concept that atrial and ventricular arrhythmias in BrS may arise from a common electrophysiological substrate rather than representing independent phenomena.

### 4.1. Atrial Fibrillation and miRNAs Expression

The finding that miR-138-5p was significantly reduced in exosomes from BrS patients, while miR-206 showed a similar but non-significant trend, further supports the presence of altered regulatory mechanisms involving the CNP pathway.

Mechanistically, atrial fibrillation may favor ventricular arrhythmogenesis through shortening of refractory periods, rapid ventricular activation, and short–long–short sequences. However, previous reports have shown persistence of ventricular arrhythmias even after AF rhythm control, suggesting that AF may be a marker of a broader arrhythmogenic substrate rather than its sole trigger [41].

Both miR-138-5p and miR-206 have been implicated in atrial electrophysiology, structural remodeling, and arrhythmogenic susceptibility [38,40,42]. A recent study [38] demonstrated that miR-138-5p expression was significantly reduced in patients with AF and that this miRNA inhibits cardiomyocyte proliferation, a process involved in fibrotic remodeling. Experimental data indicate that miR-206 contributes to arrhythmogenesis by downregulating Connexin-43, a gap-junction protein central to the pathophysiology of AF [43]. In a canine model, miR-206 was also linked to AF initiation by targeting GTP cyclohydrolase I, thereby influencing cardiac autonomic remodeling and reducing AF susceptibility [44]. In our patients, a reduction in the exosomal export of these miRNAs may therefore indicate dysregulation of post-transcriptional processes relevant to atrial and ventricular excitability.

This interpretation is consistent with evidence that atrial and ventricular arrhythmias may share overlapping molecular mechanisms in BrS [45,46].

### 4.2. Importance of Exosomal Export of miRNAs in Brugada Syndrome

In contrast, miR-142-5p, miR-223-5p, and miR-4443 displayed comparable exosomal levels between BrS patients and controls, suggesting that only a subset of *CNP*-associated miRNAs is selectively altered in this condition. The positive correlation identified between exosomal *CNP* and miR-4443 is particularly noteworthy. Although miR-4443 did not differ between groups, its association with exosomal *CNP* may indicate shared regulatory pathways or coordinated export mechanisms.

CNP signaling may be particularly relevant in this setting. Activation of NPR-B increases intracellular cGMP, which can stimulate phosphodiesterase-2 while inhibiting phosphodiesterase-3, thereby modulating cyclic nucleotide balance and β-adrenergic signaling [47,48]. Through these pathways, CNP may influence atrial electrophysiology and susceptibility to AF.

Notably, miR-4443 has been linked to inflammatory and fibrotic processes [40] and was shown to play a key role in AF pathogenesis [49], where it inhibited fibroblast proliferation, migration, invasion, and myofibroblast transformation, while promoting apoptosis through the regulation of thrombospondin-1 and downstream TGF-β1/α-SMA/collagen signaling. In our study, this correlation may therefore reflect subtle remodeling processes at the molecular level, potentially preceding overt electrophysiological or structural abnormalities. Moreover, the extensive pattern of significant correlations observed among the analyzed miRNAs suggests the presence of a coordinated regulatory network rather than isolated molecular changes.

Given their ability to simultaneously regulate multiple target genes, miRNAs may orchestrate broader disease phenotypes involving fibrosis, inflammation, ion-channel regulation, and structural remodeling [50,51].

In particular, the strong association between miR-138-5p and miR-206, together with their parallel trend toward reduction, supports the hypothesis that these miRNAs may be controlled by common regulatory signals or preferentially packaged together into exosomes. Similarly, the correlations involving miR-223-5p, miR-142-5p, and miR-4443 may reflect interconnected pathways related to inflammation and fibrotic remodeling, processes known to contribute to arrhythmogenic substrates. Taken together, these findings suggest that in BrS, exosomes portray disease-related alterations in *CNP* mRNA expression and miRNA regulatory networks. The increase in exosomal *CNP* mRNA in BrS may represent a compensatory response aimed at modulating cGMP-dependent signaling or counterbalancing enhanced adrenergic activity, both mechanisms central to atrial and ventricular arrhythmogenesis. However, given the exploratory nature of this study, these interpretations should be considered hypothesis-generating rather than mechanistically conclusive. Moreover, the selective downregulation of miR-138-5p, a miRNA involved in ion-channel regulation, fibrosis, and atrial electrophysiology [38,42,43,44], may contribute to the heightened susceptibility of BrS patients to atrial fibrillation, reinforcing the concept of a shared molecular substrate between atrial and ventricular arrhythmias in this syndrome.

### 4.3. Importance of Exosome Analysis for Early Remodeling and Arrhythmogenic Substrate

Overall, our study demonstrates that exosomal *CNP* and selected *CNP*-related miRNAs are altered in BrS, suggesting modifications in molecular packaging that may reflect early pathophysiological remodeling, namely subtle changes in gene-regulatory and signaling pathways occurring before overt structural abnormalities or clinically evident arrhythmias become detectable. These alterations may involve intercellular communication, ion-channel regulation, fibrosis-related pathways, and cellular stress responses.

These findings highlight that exosomal analysis may provide a more sensitive and biologically meaningful approach to assessing molecular remodeling in BrS than conventional blood-based measurements. The differential distribution of *CNP* and its associated miRNAs across biological compartments underscores the importance of evaluating biomarker localization, rather than solely expression levels.

However, the main limitation of this study is the relatively small sample size, which may reduce the statistical power and robustness of the conclusions. This limitation also reflects the rarity and specific nature of Brugada Syndrome, making the enrollment of a large number of patients particularly challenging. Therefore, the present findings should be considered preliminary and hypothesis-generating.

## 5. Conclusions

This study provides initial evidence that exosomal *CNP* mRNA and selected *CNP*-related miRNAs are altered in patients with Brugada Syndrome, supporting the presence of early molecular remodeling not captured by conventional blood-based analyses. These findings highlight the potential of exosomal profiling as a sensitive and biologically meaningful approach for investigating disease-associated signaling pathways. Future studies involving larger and independently validated patient cohorts, dedicated cellular models, and mechanistic functional assays will be crucial to clarify whether these exosomal alterations contribute directly to BrS pathogenesis or reflect compensatory responses within a broader arrhythmogenic substrate.

This may be particularly relevant in BrS, where atrial and ventricular arrhythmias frequently coexist and may reflect shared molecular remodeling pathways.

Elucidating this distinction may ultimately pave the way for improved diagnostic biomarkers, personalized risk stratification, and targeted therapeutic interventions.

## Figures and Tables

**Table 1:** Mature miRNA sequence and *CNP* oligonucleotides.

Gene	Forward Primer Sequence (5′—3′)	GenBank, Accession Number	Location	Ta, °C
***CNP***	Hs_*NPPC*_2_SG QuantitectPrimerAssay, QIAGEN (blind)	NM_024409	chr 2q37.1	55 °C
**hsa-miR-223-5p**	CGTGTATTTGACAAGCTGAGTT	LM608368	chr Xq12	55 °C
**hsa-miR-142-5p**	CATAAAGTAGAAAGCACTACT	NR_029683	chr 17q22	55 °C
**hsa-miR-138-5p**	AGCTGGTGTTGTGAATCAGGCCG	NR_029700.1	chr 3p21.32	55 °C
**hsa-miR-4443**	TTGGAGGCGTGGGTTTT	NR_039645.1	chr 3p21.31	55 °C
**hsa-miR-206**	TGGAATGTAAGGAAGTGTGTGG	NR_029713.1	chr 6p12.2	55 °C

**Table legend. *CNP*:** or *NPPC*, C-type natriuretic peptide; **hsa-miR-223-5p:** homo sapiens miRNA-223 with 5p strand present in the forward position; **hsa-miR-142-5p:** homo sapiens miRNA -142 with 5p strand present in the forward position; **hsa-miR-138-5p:** homo sapiens miRNA -138a with 5p strand present in the forward position; **hsa-miR-4443:** homo sapiens miRNA -4443; **hsa-miR-206:** homo sapiens-miRNA 206.

**Table 2:** Clinical characteristics of controls and patients with Brugada syndrome.

	Controls	BrS Patients	*p*
***ANTHROPOMETRIC VARIABLES***			
Sex, Male	9/14	12/18	ns
Age, years	40.5 ± 3.2	47.6 ± 3.0	ns
Smoke	0/14	84/12	ns
***METABOLIC VARIABLES***			
Type 2 diabetes mellitus	0/14	1/18	ns
Dyslipidemia	2/14	1/18	ns
Weight, kg	68.3 ± 3.3	74.0 ± 3.5	ns
Height, cm	171.4 ± 2.2	170.3 ± 2.2	ns
Body Mass Index	23.0 ± 0.7	25.3 ± 0.7	0.03
***CLINICAL VARIABLES***			
Sudden death familiarity	3/14	2/18	ns
Family History of BrS	0/14	6/18	0.008
pathogenic/likely pathogenic SCN5A variant	0/14	4/18	ns
Pre-syncope episodes	2/14	7/18	ns
Hypertension	0/14	5/18	0.002
Heart rate, bpm	64.6 ± 2.2	64.5 ± 3.3	ns
Implantable cardioverter-defibrillator	0/14	6/18	0.008
Ventricular extrasystole	7/14	0/18	0.002

Data are presented as mean ± SEM. ns = not significant.

**Table 3:** *CNP* (alias *NPPC*) miRNA targets found using microT-CDS algorithm and ranking following miTG score threshold at 0.7 prediction (MicroT-CDS, DIANA tool).

Transcript ID	miRNA Name	miRNA Name	miRNA Name
***NPPC/CNP***	hsa-miR-1277-5p	hsa-miR-374b-5p	hsa-miR-3689d
	hsa-miR-548x-3p	hsa-miR-3163	hsa-miR-1250-3p
	hsa-miR-410-3p	hsa-miR-3121-3p	hsa-miR-147a
	hsa-miR-548aj-3p	hsa-miR-548f-3p	***hsa-miR-142-5p***
	hsa-miR-6780b-3p	hsa-miR-369-3p	hsa-miR-3133
	hsa-miR-5692c	hsa-miR-548az-3p	hsa-miR-374a-5p
	hsa-miR-5692b	hsa-miR-548e-3p	***hsa-miR-4443***
	hsa-miR-6076	hsa-miR-579-3p	hsa-miR-3180-5p
	***hsa-miR-138-5p***	hsa-miR-664b-3p	hsa-miR-599
	hsa-miR-3908	hsa-miR-1258	hsa-miR-449b-3p
	hsa-miR-340-5p	hsa-miR-338-5p	hsa-miR-623
	hsa-miR-5011-5p	hsa-miR-548ac	hsa-miR-3120-3p
	hsa-miR-323a-3p	hsa-miR-548z	hsa-miR-133a-5p
	hsa-miR-6505-3p	hsa-miR-548h-3p	hsa-miR-495-3p
	hsa-miR-6797-3p	hsa-miR-153-5p	hsa-miR-4697-3p
	hsa-miR-548am-3p	hsa-miR-6884-5p	hsa-miR-4495
	hsa-miR-548ah-3p	hsa-miR-6851-5p	*hsa-miR-33a-3p*
	hsa-miR-548aq-3p	hsa-miR-190a-3p	hsa-miR-548g-3p
	hsa-miR-548ae-3p	hsa-miR-4753-3p	hsa-miR-374c-5p
	hsa-miR-548j-3p	hsa-miR-5696	***hsa-miR-223-5p***
	hsa-miR-5692a	hsa-miR-1197	hsa-miR-4803
	hsa-miR-6867-5p	hsa-miR-770-5p	hsa-miR-1-3p
	hsa-miR-4282	hsa-miR-548d-3p	***hsa-miR-206***
	hsa-miR-4729	hsa-miR-6516-3p	hsa-miR-548q
	hsa-miR-335-3p	hsa-miR-548bb-3p	hsa-miR-586
	hsa-miR-1207-3p	hsa-miR-491-3p	hsa-miR-5680

## Data Availability

The original contributions presented in this study are included in the article. Further inquiries can be directed to the corresponding author.

## Abbreviations

The following abbreviations are used in this manuscript:

BrS	Brugada Syndrome
AF	Atrial fibrillation
CNP	C-type natriuretic peptide
miRNAs/miR	microRNAs
β-AR	β-adrenergic receptor
ddPCR	Droplet Digital™ PCR
NPR-B	Natriuretic peptide receptor B
GC	guanylyl cyclase
PDE2	phosphodiesterase 2
PDE3	phosphodiesterase 3
3′UTR	3′ untranslated region
